# Prioritizing key synergistic circulating microRNAs for the early diagnosis of biliary tract cancer

**DOI:** 10.3389/fonc.2022.968412

**Published:** 2022-10-06

**Authors:** Fei Su, Ziyu Gao, Yueyang Liu, Guiqin Zhou, Wei Gao, Chao Deng, Yuyu Liu, Yihao Zhang, Xiaoyan Ma, Yongxia Wang, Lili Guan, Yafang Zhang, Baoquan Liu

**Affiliations:** ^1^ College of Bioinformatics Science and Technology, Harbin Medical University, Harbin, China; ^2^ Department of Anatomy, Harbin Medical University, Harbin, China; ^3^ Department of Immunology, Harbin Medical University, Harbin, China; ^4^ Laboratory of Medical Genetics, Harbin Medical University, Harbin, China; ^5^ Department of Information Management, Shanghai Lixin University of Accounting and Finance, Shanghai, China; ^6^ Department of Modern Medicine and Pharmacy, University of Tibetan Medicine, Lhasa, China

**Keywords:** synergistic, circulating microRNAs, noninvasive biomarker, diagnosis, biliary tract cancer

## Abstract

Biliary tract cancer (BTC) is a highly aggressive malignant tumor. Serum microRNAs (ser-miRNAs) serve as noninvasive biomarkers to identify high risk individuals, thereby facilitating the design of precision therapies. The study is to prioritize key synergistic ser-miRNAs for the diagnosis of early BTC. Sampling technology, significant analysis of microarrays, Pearson Correlation Coefficients, t-test, decision tree, and entropy weight were integrated to develop a global optimization algorithm of decision forest. The source code is available at https://github.com/SuFei-lab/GOADF.git. Four key synergistic ser-miRNAs were prioritized and the synergistic classification performance was better than the single miRNA’ s. In the internal feature evaluation dataset, the area under the receiver operating characteristic curve (AUC) for each single miRNA was 0.8413 (hsa-let-7c-5p), 0.7143 (hsa-miR-16-5p), 0.8571 (hsa-miR-17-5p), and 0.9365 (hsa-miR-26a-5p), respectively, whereas the synergistic AUC value increased to 1.0000. In the internal test dataset, the single AUC was 0.6500, 0.5125, 0.6750, and 0.7500, whereas the synergistic AUC increased to 0.8375. In the independent test dataset, the single AUC was 0.7280, 0.8313, 0.8957, and 0.8303, and the synergistic AUC was 0.9110 for discriminating between BTC patients and healthy controls. The AUC for discriminating BTC from pancreatic cancer was 0.9000. Hsa-miR-26a-5p was a predictor of prognosis, patients with high expression had shorter survival than those with low expression. In conclusion, hsa-let-7c-5p, hsa-miR-16-5p, hsa-miR-17-5p, and hsa-miR-26a-5p may act as key synergistic biomarkers and provide important molecular mechanisms that contribute to pathogenesis of BTC.

## 1 Introduction

Biliary tract cancer (BTC) is a highly aggressive malignant tumor, and its morbidity has increased in recent years; however, the underlying pathogenetic mechanism and predisposing factors remain unclear (1). Traditional surgical puncture biopsy is a complex and invasive procedure that can cause physical and psychological discomfort in patients (2). Therefore, developing a non-invasive method to identify specific molecular markers for the early detection of BTC is an urgent need. Pancreatic cancer (PC) is the fourth leading cause of cancer death in Europe and the United States, and the overall 5-year survival rate is approximately 7% (3). Because of the close anatomical position and similar early symptoms, as well as similar microRNA (miRNA) expression profiles of BTC and PC (4), patients with an equivocal diagnosis might not receive optimum treatment and the expected results. Biomarkers capable of differentiating BTC from PC are important to distinguish the two kinds of cancer (5). Therefore, developing accurate and reliable biomarkers for BTC and PC is an important clinical issue.

MiRNAs are a class of short (18–22 nucleotides) evolutionarily conserved, non-coding RNAs that play critical roles in diverse physiological and pathological processes (6). Because ideal biomarkers should be non-invasive, low cost, and be detected easily, circulating miRNAs have natural advantages (7). Circulating miRNAs are renewable and consistent in serum or plasma. They are non-invasive biological markers and are characterized by convenient detection, which makes them promising biomarkers for monitoring and early diagnosis (8). Circulating miRNA expression is frequently dysregulated in malignant tumors (9). In previous work from our group, we showed that the expression of ser-miR-21 is significantly correlated with the efficacy of combination treatment with neoadjuvant chemotherapy and trastuzumab and with survival in HER2-positive breast cancer patients (10). Another result of our study showed that ser-miR-34a serves as a biomarker for predicting the treatment response and prognosis of neoadjuvant chemotherapy in breast cancer (11). An increasing number of circulating miRNAs that are dysregulated in BTC were identified recently (12–14). Downregulation of ser-miR-106a is a powerful prognostic indicator for BTC patients, and it is a lymph node metastasis indicator. High levels of ser-miR-21, a diagnostic and prognostic biomarker in BTC, are related to adverse clinical features, diminished survival, and poor prognosis (15, 16). Plasma miR-21 levels are higher in early BTC patients than in healthy volunteers, suggesting that it could be used as a biomarker for BTC (17). Serum analysis revealed that decreased ser-miR-106a is associated with a higher likelihood of lymph node metastasis, which results in poor outcomes in BTC patients (18). Analysis of the expression of circulating miRNAs in gallbladder cancer patients showed a significant relationship between the differential expression miR-187, miR-143, and miR-202 and tumor-node-metastasis stage (17).

The synergistic effects of miRNAs on cancer were shown previously (19–22). We screened serum miR-21 and miR-125b as markers predicting the response to neoadjuvant chemotherapy and prognosis in stage II/III breast cancer (23, 24). A prognostic model based on four circulating miRNAs predicted relapse and survival in diffuse large B-cell lymphoma (24). A panel of six circulating miRNAs in serum showed a strong potential as a diagnostic biomarker signature for patients with colorectal carcinoma (25). However, there are no studies prioritizing circulating miRNAs acting synergistically for the diagnosis of BTC. In this study, we proposed the use of the Global Optimization Algorithm of Decision Forest to predict novel sensitive, reliable, and noninvasive synergistic ser-miRNAs for the diagnosis of BTC by integrating Significant Analysis of Microarrays, Pearson Correlation Coefficient, t-test, decision tree, and entropy weight. The results showed that the synergistic classified performance of the decision tree including hsa-let-7c-5p, hsa-miR-16-5p, hsa-miR-17-5p, and hsa-miR-26a-5p was more reliable than that of single miRNAs for discriminating BTC patients from healthy controls and discriminating BTC from early PC (26). The target genes were significantly associated with cell cycle sub-pathways. In Protein-Protein Interaction (PPI) Network, TP53 was the most crucial gene with the highest connectivity degree, which was associated with poor clinicopathologic characteristics and survival in patients with BTC. In addition, hsa-miR-26a-5p was a negative prognostic factor in BTC, patients with high expression had shorter survival than those with low expression. The results suggested that the proposed method could provide key clinical markers for early diagnosis of cancer.

## 2 Materials and methods

### 2.1 Data collection

Human serum BTC microarray datasets from Gene Expression Omnibus (GEO, https://www.ncbi.nlm.nih.gov/geo/) were used in this study. The internal dataset (GSE85589) included 101 BTC patients and 19 healthy controls. The external dataset (GSE59856) included 98 BTC patients and 150 healthy controls. If multiple probes matched to one miRNA, the average expression value of the probe was considered as the expression of the miRNA. There were 62277 pairs of regulatory relationships between 850 human miRNAs and 15841 target genes, of which 829 pairs were from mir2Disease (5), 1798 pairs were from miRecords (27), 39079 pairs were from miRTarBase (28), and 20571 pairs were from DIANA-TarBase_V8 (29).

### 2.2 Global optimization algorithm of the decision forest

Global Optimization Algorithm of the Decision Forest (GOADF) contained sampling technology, integrating the decision forest, prioritizing the candidate ser-miRNA signatures by decision tree entropy weight score algorithm.

#### 2.2.1 Sampling internal training sets and internal test sets

**Figure 1:**
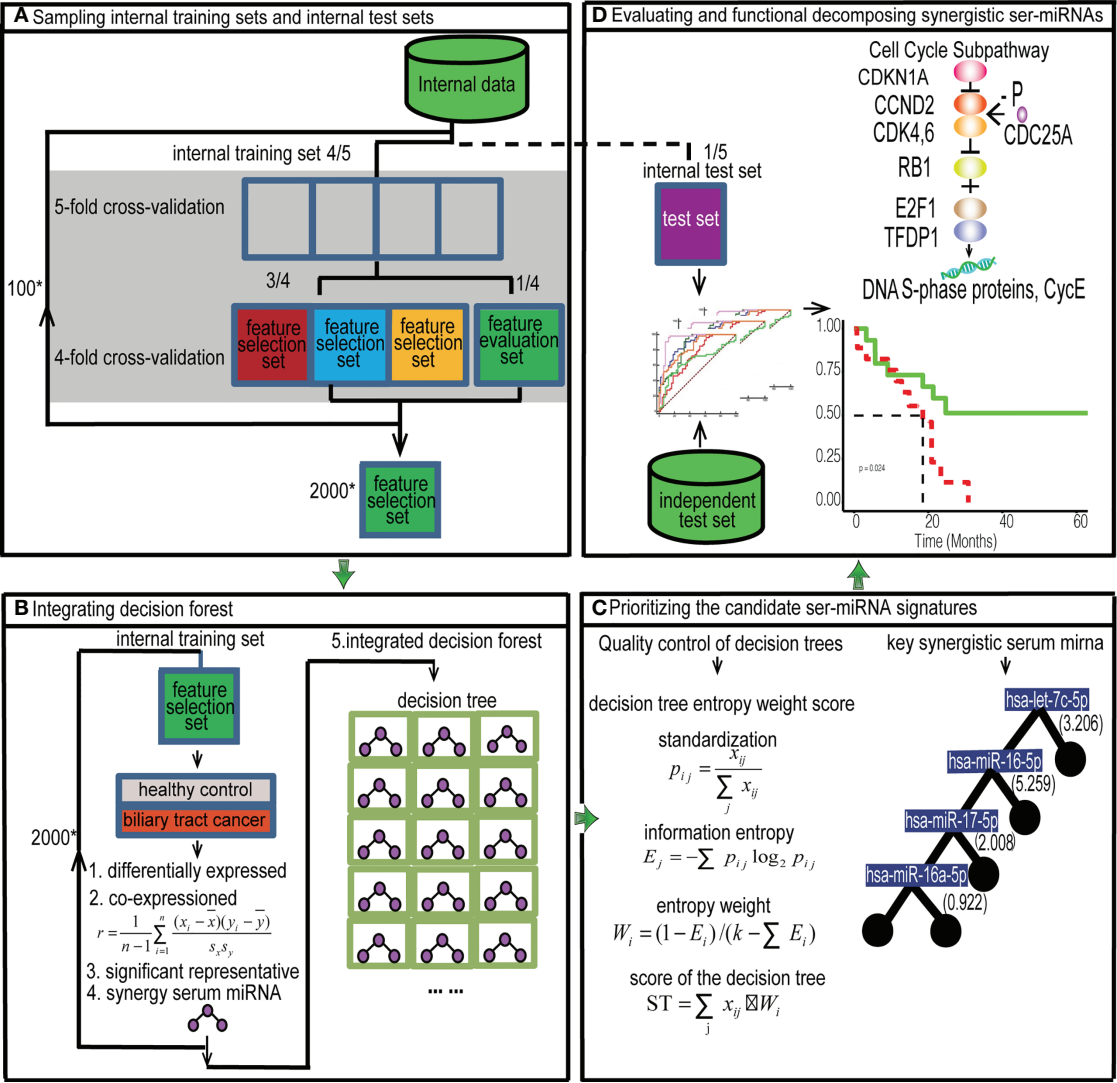
Flow chart. Prioritizing synergistic circulating miRNAs for the early diagnosis of BTC by GOADF. **(A)** The internal data set was divided into the feature selection dataset, feature evaluation dataset and internal test set and 2,000 feature selection sets were produced by sampling technology. **(B)** In internal training set, every feature selection set produced a decision tree. The 2,000 decision trees were integrated into decision forest. **(C)** We filtered out the trees with low quality and sorted the decision tree by ST to identify the key synergistic ser-miRNAs. **(D)** The synergistic ser-miRNAs were evaluated in the internal and independent test sets. The function of synergistic ser-miRNAs was to decompose by KEGG sub-pathway identification and GO enrichment. ”*” means multiple relationship.

The internal dataset was divided into five subsets of equal size randomly as shown in the flow chart (**Figure 1A**). Four subsets were used as the internal training dataset to train the classifier, and one subset was used as the internal test dataset to evaluate the classifier (5-fold cross-validation). Sampling was repeated by using each subset as the feature test dataset in turn, and the optimized classifier was evaluated on the internal test set. To explore the classification performance of the classifier, the training set was divided into four equal-sized subsets randomly, of which three subsets were used as the feature selection dataset to build the classifier and one subset was used as the feature evaluation dataset (4-fold cross-validation) to select the classifier with the best classification performance. Sampling was repeated by using each subset as the feature evaluation dataset in turn to select the optimized classifier. A sampling will produce 20 feature selection sets, and we sampled 100 times at random to produce 2000 feature selection sets.

#### 2.2.2 Integrating the decision forest

For every feature selection set (**Figure 1B**), SAM (P< 0.05) was used to identify the differentially expressed ser-miRNAs. Then, the differentially expressed ser-miRNAs were clustered into co-expressed serum miRNA sets by PCC (r > 0.6).

Pearson Correlation Coefficient is example 1 of an equation:


(1) $$r=\frac{1}{n-1}\sum_{i=1}^{n}\frac{\left(x_{i}-\bar{x})(y_{i}-\bar{y}\right)}{S_{x}S_{y}}$$

*n*: number of samples, x_i_: expression levels of ser-miRNAi, y_i_: expression levels of ser-miRNAj, $\bar{x}$ : average expression level of ser-miRNAi, $\bar{y}$ : average expression level of ser-miRNAj, *s_x_*: standard deviation of ser-miRNAi, *s_y_*: standard deviation of ser-miRNAj.

For each co-expressed ser-miRNA set, the t-test (P< 0.01) was used to identify key miRNAs. To obtain the global optimal ser-miRNAs, the significant representative miRNAs in all co-expressed sets of a feature selection set were used to construct a decision tree classifier, in each tree, the serum miRNAs were selected to ensure that they led to maximal purity at the nodes of each branching, and this binary tree was partitioned until the growth was stopped. The synergistic miRNAs were extracted for each tree.

For each feature selection set, a decision tree classifier was established. There were 2000 feature selection sets in the internal training set, and 2000 decision trees were produced and the decision trees were integrated into a decision forest.

#### 2.2.3 Prioritizing the candidate Ser-miRNA signatures

The first step was quality control of decision trees (**Figure 1C**). The trees with duplicated miRNAs and those with< 3 miRNAs were removed from the internal evaluation sets. The trees were retained if they had a AUC of single miRNA > 0.7, average frequency of miRNAs greater than the average frequency of miRNAs in the forest, and accuracy, sensitivity, and specificity > 0.5.

The second step is decision tree entropy weight score algorithm. Data standardization was necessary, assuming that the importance of the tree was related to *k* factors: X_1_, X_2_,…,X_k_, for the *i* th tree, the effect factors were *x*_i1_,*x_i2_,…x_ik_*. Of these, *x_ij_* denoted the value of the *j* th factor for the *i* th tree. Assuming that the standardized value of each factor was *p_ij_*, this is example 2 of an equation:


(2) $$P_{ij}=\frac{x_{ij}}{\sum_{j}x_{ij}}$$

According to the definition of information entropy, the information entropy of the *j*th element was example 3 of an equation:


(3) $$E_{j}=\sum_{j}p_{ij}\log_{2}p_{ij}$$

If *p_ij_* = 0, then $\underset{p_{ij}\to 0}{\lim}p_{ij}\log_{2}p_{ij}=0$

The basic notion of entropy weight consists of determining the importance of factors according to the variation of factors. In general, if the information entropy of a factor is smaller, the variation degree of the factor is greater; the more information it provides, the greater a role it could play in the comprehensive evaluation and the greater its weight. On the contrary, if the information entropy of a factor is larger, the variation degree of the index value factor is smaller; the smaller its role in the comprehensive evaluation, the smaller its weight.

The formula 4 for determining entropy weight is as follows:


(4) $$W_{i}=(1-Ei)/k-\sum Ei)(1,2,3,\text{...}k)$$

k is the number of factors.

To select the decision tree in the forest, the equation 5 of Score of Trees (ST) was determined based on the entropy weight.


(5) $$ST=\sum_{j}x_{ij}*W_{i}$$

Five factors were important for the *i*th tree, namely, the average frequency of miRNAs in the decision forest, the synergistic AUC value, accuracy, sensitivity, and specificity in the internal feature evaluation set. Then, the tree was ranked according to the ST value in decreasing order, and the tree with the highest ST score was obtained and used as the classifier. The miRNAs on the tree were used as the candidate ser-miRNA features.

### 2.3 Evaluation of ser-miRNAs

The performance of the ser-miRNAs was evaluated in the internal test set and independent test set (**Figure 1D**). The classification performance was assessed by calculating the AUC of single ser-miRNA, and the AUC, accuracy, sensitivity, specificity, and F-measure of synergetic ser-miRNAs.

Sub-pathway enrichment analysis of target genes of the decision tree classifier was performed by iSubpathwayMiner R package and the cutoff was P< 0.01 (14, 30). GO enrichment analysis was performed by clusterProfiler R package and the cutoff was P< 0.01 (18, 31).

### 2.4 Survival analysis

To identify the ser-miRNAs that could predict BTC patient survival, we identified two ser-miRNAs (hsa-miR-26a-5p, hsa-miR-16-5p) that map to the BTC tissues of TCGA (https://xenabrowser.net/datapages/). Univariate Cox regression analysis was used to evaluate the association between survival and the expression level of each ser-miRNA. All BTC patients in TCGA were thus assigned to high- risk and low-risk groups using the median risk score as the cut-off point. The Kaplan-Meier method was used to estimate the overall survival time for the two subgroups, and differences in survival time were analyzed using the log rank test. All analyses were performed using R 3.6.3 statistical software.

## 3 Results

### 3.1 A decision forest for prioritizing key synergistic ser-miRNAs

A total of 2000 feature selection sets were produced using the sampling method, and each feature selection set produced a tree. We integrated a decision forest including 2000 decision trees. After filtering out the decision trees with duplicated miRNAs and the trees with< 3 miRNAs, 542 trees were obtained (**Table S1**). In addition, 41 decision trees were obtained with the AUC of single miRNA in the internal evaluation set > 0.7 (**Table S2**), of which 19 trees had an average frequency of miRNAs above the average in the decision forest (221.2051). Then, 13 trees with accuracy, sensitivity, and specificity > 0.5 were identified. Then GOADF was used to score the trees according to ST. Finally, the tree S16L28 with the highest ST (0.8597) was selected (**Table S3**), S16 represented the 16th feature selection set, and L28 represented the 28th loop.

To further investigate the significant of the ST value of the decision tree, we randomly shuffled the sample labels of ser-miRNA expression profile, while keeping the number of ser-miRNA and sample unchanged. The actual ST value was significantly higher (P=0.0003) than the ST value of the 3,426 randomly shuffled decision trees. This result revealed that GOADF could identify key synergistic ser-miRNAs.

### 3.2 Key synergistic ser-miRNAs serve as biomarkers for BTC

**Figure 2:**
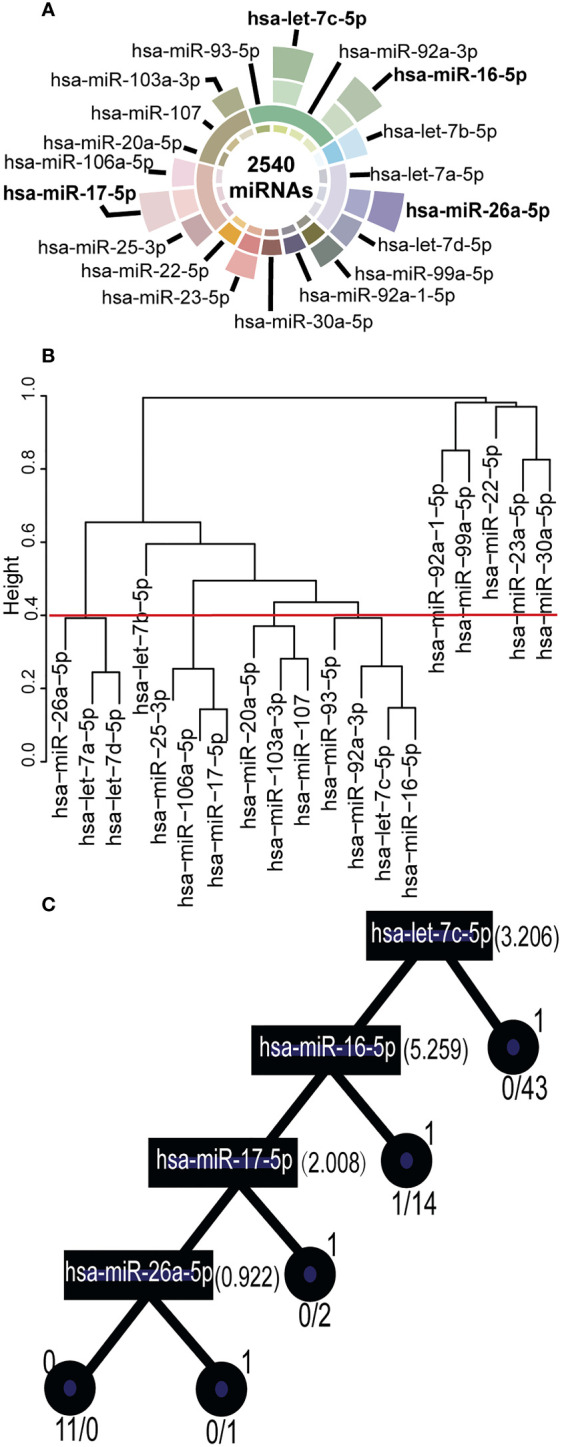
Key Synergistic Ser-miRNAs Serve as Biomarkers for BTC. **(A)** Filtered key synergistic ser-miRNAs. The center of a circle represented 2,540 ser-miRNAs in the internal data sets. From the center of the circle to the outside, the first layer represented 19 differentially expressed ser-miRNAs that were identified by SAM. The second layer expressed the 10 co-expression ser-miRNA set using PCC. The third layer expressed 11 key ser-miRNAs that were selected using a t-test. The outermost layer represented four key synergistic ser-miRNAs that were identified by decision tree. **(B)** Clustered into co-expressed ser-miRNA sets by PCC. The 19 differentially expressed ser-miRNAs were clustered into 10 co-expressed ser-miRNA sets by PCC (r>0.6, P<0.05). **(C)** A decision tree was generated to further select the key synergistic ser-miRNAs. The squares represented ser-miRNAs: hsa-let-7c-5p, hsa-miR-16-5p, hsa-miR-17-5p, and hsa-miR-26a-5p and the split value was marked on the right. The circles represented the classification status of the samples, 1 represented BC samples, 0 represented healthy control samples.

The filtering process for ser-miRNAs on the tree S16L28 is following (**Figure 2**; **Table S4**). First, there were 2,540 miRNAs in the training set, among which 19 differentially expressed ser-miRNAs in BTC were identified by SAM (P< 0.05). Then, these differentially expressed ser-miRNAs were clustered into 10 co-expressed ser-miRNA sets by PCC (r>0.6, P<0.05). Key ser-miRNAs were selected in each co-expressed ser-miRNA set by t-test (P< 0.01) and 11 key ser-miRNAs were identified: hsa-let-7d-5p, hsa-miR-26a-5p, hsa-let-7b-5p, hsa-let-7c-5p, hsa-miR-16-5p, hsa-miR-103a-3p, hsa-miR-17-5p, hsa-miR-106a-5p, hsa-miR-25-3p, hsa-miR-23a-5p, and hsa-miR-99a-5p. A decision tree was generated to further select four key synergistic ser-miRNAs, including hsa-let-7c-5p, hsa-miR-16-5p, hsa-miR-17-5p, and hsa-miR-26a-5p.

To further investigate the significance of tree S16L28, we compared the average frequencies of miRNA on this tree with the 3,426 randomly shuffled decision trees. The actual frequency was 281, which was significantly higher (P=0.0001) than the ST average frequency of the 3,426 randomly shuffled decision trees. This result revealed that GOADF could identify key synergistic ser-miRNAs as biomarkers.

### 3.3 Key synergistic ser-miRNAs improve the classification performance for early BTC in the internal data set

**Figure 3:**
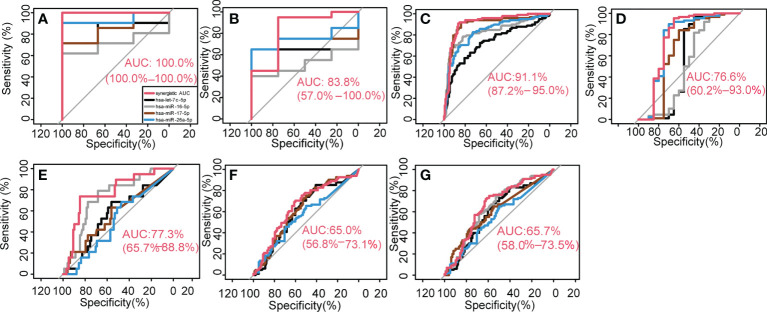
Key synergetic ser-miRNAs classification performance. They classified BTC from healthy controls in the internal data **(A, B)**, external independent test dataset **(C)** and classified BTC from the PC **(D–G)**.

In the internal feature evaluation set, the key synergistic ser-miRNAs were used to classify samples from BTC patients and healthy controls. The single AUC was 0.8413, 0.7143, 0.8571, and 0.9365 for hsa-let-7c-5p, hsa-miR-16-5p, hsa-miR-17-5p, and hsa-miR-26a-5p, respectively, whereas the synergistic AUC was 1.00 (95% confidence interval (CI) ci: 100%–100%, P< 0.001) (**Figure 3A**), which was bigger than the largest AUC of the single miRNA (0.95) among the 13 trees. The accuracy of synergistic ser-miRNAs was 1, sensitivity was 1, specificity was 1, and F-measure was 1.

In the internal test set the AUC of each single biomarker was 0.6500, 0.5125, 0.6750, and 0.7500 for hsa-let-7c-5p, hsa-miR-16-5p, hsa-miR-17-5p, and hsa-miR-26a-5p respectively, however, four key synergistic ser-miRNAs increased the AUC value to 0.8375 (95% ci:57%–100%, P< 0.001) (**Figure 3B**), and the accuracy of synergistic ser-miRNAs was 0.9167, sensitivity was 0.9500, specificity was 0.7500, and F-measure was 0.9500.

To further investigate the classification performance of key synergistic ser-miRNAs, the synergistic AUC values of ser-miRNAs were significantly higher (P=0.0001) than the synergistic AUC values of the 3,426 randomly-shuffled decision trees in the internal feature evaluation set. These results revealed a high classification performance of key synergistic ser-miRNAs.

### 3.4 Key synergistic ser-miRNAs improve the classification performance for early BTC detection in the independent test set

An independent test dataset (GSE59856) was used to verify the classification effectiveness of key synergistic ser-miRNAs. The classification performance of the four ser-miRNAs was assessed. The AUCs of hsa-let-7c-5p, hsa-miR-16-5p, hsa-miR-17-5p, and hsa-miR-26a-5p were 0.7279, 0.8313, 0.8957, and 0.8303, respectively, whereas the synergistic actions of ser-miRNAs increased the AUC values to 0.9110 (95% ci: 87.2%–95.0%, P< 0.001) for discriminating between BTC patients and health controls (**Figure 3** **C**). This result confirmed that the key synergistic ser-miRNAs served as robust biomarkers for diagnosis of BTC. The key synergistic ser-miRNAs were closely related to the diagnosis of BTC and validated the effectiveness of the GOADF method.

To further investigate the synergistic action of key synergistic ser-miRNAs, the synergistic AUC value of ser-miRNAs was significantly higher (P=0.0496) than the synergistic AUC values of the 3,426 randomly shuffled decision trees in the independent test set. These results revealed a high classification performance of key synergistic ser-miRNAs.

### 3.5 Key synergistic ser-miRNAs serve as novel powerful biomarkers for distinguishing BTC from PC

A decision tree was used to distinguish BTC from PC. In the internal data set, 20 BTC samples from the test set of the tree S16L28 were used as controls, and 88 PC samples were used as the case set. The AUCs of hsa-let-7c-5p, hsa-miR-16-5p, hsa-miR-17-5p, and hsa-miR-26a-5p were 0.5619, 0.5216, 0.6608, and 0.7540, respectively. The synergistic AUC was 0.7660 (95% ci: 0.6015–0.9303, P=1.5190*10-3) (**Figure 3** **D**).

In the external independent test dataset, 98 BTC samples were used as controls and 19 early PC samples (stage II) were used as the case set. The AUCs of the four miRNAs were 0.5636, 0.7336, 0.5704, and 0.5056. The synergistic AUC was 0.7728 (95% ci: 0.6573–0.8884, P=3.6980e-6) (**Figure 3** **E**). To classify BTC and late PC, 98 BTC samples were used as controls and 81 PC samples (stages III and IV) were used as the case set. The AUCs of the four miRNAs were 0.6072, 0.6259, 0.6169, and 0.562. The synergistic AUC was 0.6497 (95% ci: 0.5678–0.7315, P =0.0003) (**Figure 3** **F**). To classify BTC and all PC patients, 98 BTC samples were used as controls and 100 PC samples (stages II, III, and IV) were used as the case set. The AUCs of the four miRNAs were 0.5989, 0.6464, 0.6081, and 0.5513 for hsa-let-7c-5p, hsa-miR-16-5p, hsa-miR-17-5p, and hsa-miR-26a-5p, respectively. The synergistic AUC was 0.657 (95% ci: 0.5797–0.7348, P=7.0024e-5) (**Figure 3** **G**). The classification performance of the four ser-miRNA for distinguishing BTC from early PC was better; however, the classification performance for distinguishing BTC from late PC was worse than that for distinguishing BTC from all PC.

### 3.6 Dissection of the function of the key synergistic ser-miRNAs in early BTC

**Figure 4:**
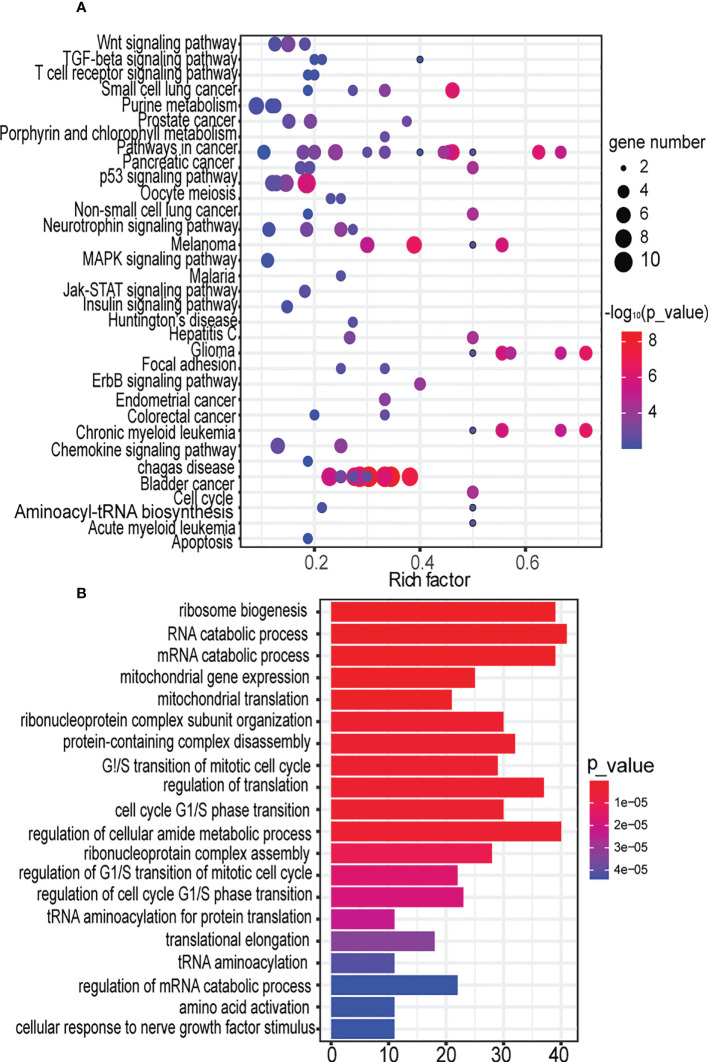
Decomposing the function of the key synergistic Ser-miRNAs in the early BTC. **(A)**. Sub-pathway enrichment analysis of target genes of the tree by KEGG. Dot plot for 104 sub-pathways signatures associated with the target genes of the tree. The y-axis represents entire pathways in KEGG where sub-pathways were located, the x-axis represents the rich factor of the pathway, which meant that the ratio of the number of genes enriched in this pathway (P<0.01) in the target gene to the number of genes belonging to this pathway in human genes. The node size indicates the gene number of the sub-pathway. The color intensity of node corresponds to the negative logarithm of the P-value based on 10. The color of the dots from blue to red indicates the significance of the sub-pathway, and the redder colors indicate more significance. **(B)**. GO analysis of target genes of trees based on their molecular functions, biological processes, and cellular components. The significant terms were shown according to the gene counts (P<0.01).

The sub-pathways regulated by ser-miRNAs were investigated. There were 589 unique target genes of tree S16L28 that were confirmed experimentally (**Table S5**). These target genes were annotated to 104 unique sub-pathways (P<0.01) belonging to 33 KEGG human pathways (**Figure 4A**; **Table S6**). Ten target genes were significantly associated with cell cycle sub-pathway 04110_23 (P=2.7627e-9). Previous studies showed that the expression of cell cycle modulating proteins, such as p16, p21, and p27, was associated with aggressive tumor behavior in several human malignancies including BTC (32), and BTC was related to disorders of major regulators of the cell cycle (33). The study demonstrated that artemisinin inhibited the proliferation of gallbladder cancer cells *in vitro* and *in vivo* and induced apoptosis by inducing cell cycle arrest. Previous studies on artemisinin showed that induction of cell cycle arrest inhibited the proliferation of BTC cells (34). Five target genes (AKT3, FOXO3, GSK3B, NFKB1, and PIK3CA) were significantly associated with a neurotrophin signaling sub-pathway (path:04722_13) P=1.670e-4), and these genes were verified in a study of survival-associated clusters in BTC (35). Another previous report indicated that high neurotrophin expression was associated with unfavorable clinicopathological findings and poor patient prognosis in BTC (17).

Next, we investigated the function of ser-miRNA targets. The target genes were significantly associated with biological process Gene Ontology (GO): 0042254, ribosome biogenesis (P=1.1700e-14) (**Figure 4B**; **Table S7**). Increased ribosome biosynthesis was closely related to the progression of blood cancers. However, this relationship was not found in BTC (36). GO analysis revealed that target genes were significantly enriched in RNA catabolic process (P=7.6000e-12). Autophagy was a catabolic process that played a context-dependent role in cancer. Autophagy may inhibit tumor initiation under specific conditions, and this observation has prompted renewed interest in targeting autophagy for cancer therapy (37).

### 3.7 Protein protein interaction network of target genes

**Figure 5:**
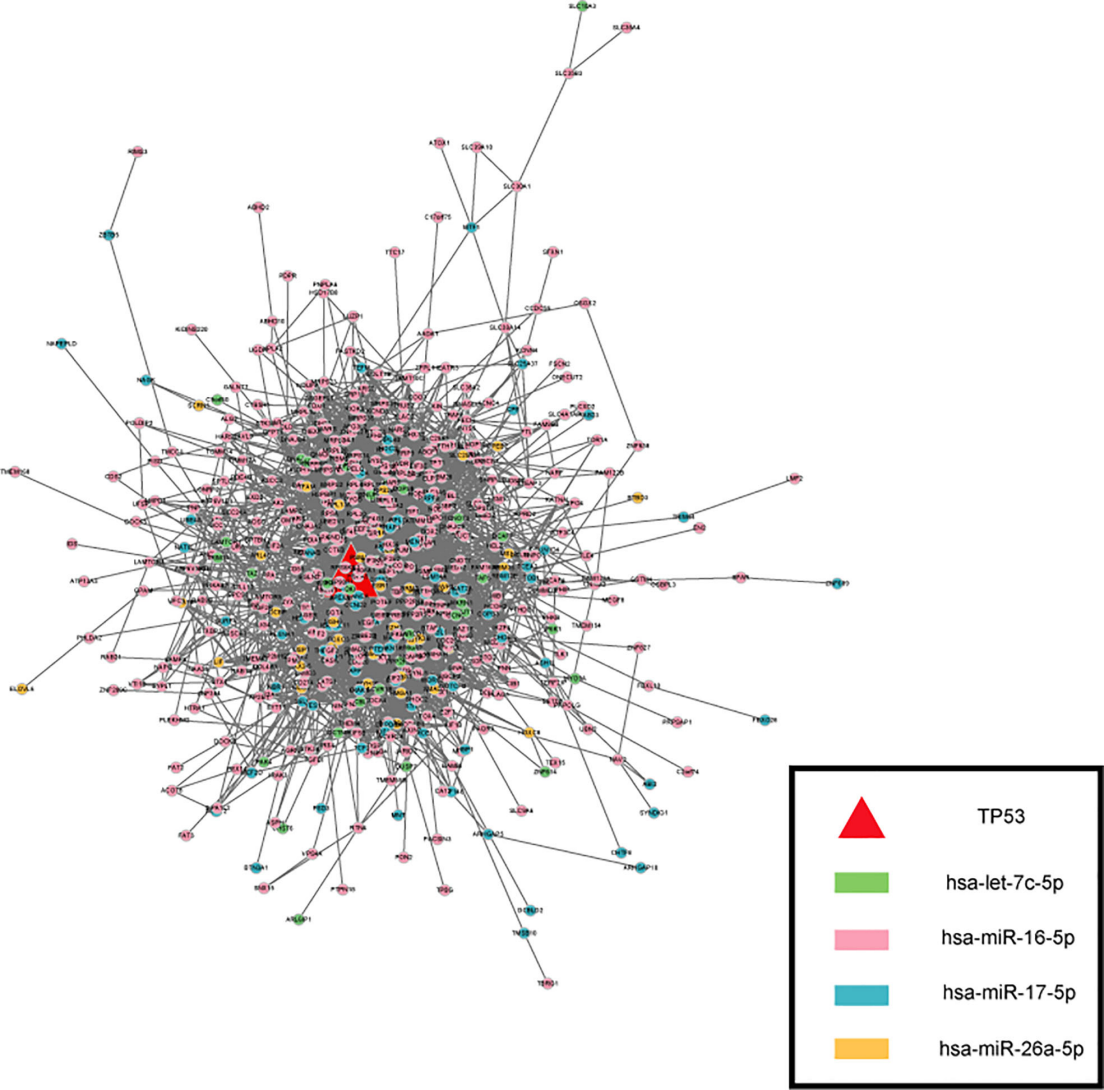
The PPI network of targets. The color is drawn according to ser-miRNA, light green, pink, dark green and yellow nodes are the targets of has-let-7c-5p、has-miR-16-5p、has-miR-17-5p and has-miR-26a-5p respectively.

Applying the STRING, the Protein Protein Interaction (PPI) network was generated (**Figure 5**), the color is drawn according to ser-miRNA. 554 nodes (genes) and 4211 edges (interactions) were established in the constructed PPI network. The top ten hub genes were identified based on their connectivity degree. The results revealed that TP53 was the most crucial gene with the highest connectivity degree=125 followed by cyclin MYC at degree=115, HSP90AA1at degree=97, PTEN at degree=76, RPL4 at degree=69, ESR1、EEF2、RPSA and EEF1G at degree=66, HSPA5 at degree=63. Previous results demonstrated that most common alterations occurred in TP53 for blood (38). TP53 mutation has been shown to be associated with poor clinicopathologic characteristics and survival in patients with BTC and TP53 mutations in BTCs are associated with enhanced gemcitabine resistance, therefore targeting TP53 may be a novel therapeutic strategy for treatment of BTC (39).

### 3.8 Key synergistic ser-miRNAs predict survival in BTC

**Figure 6:**
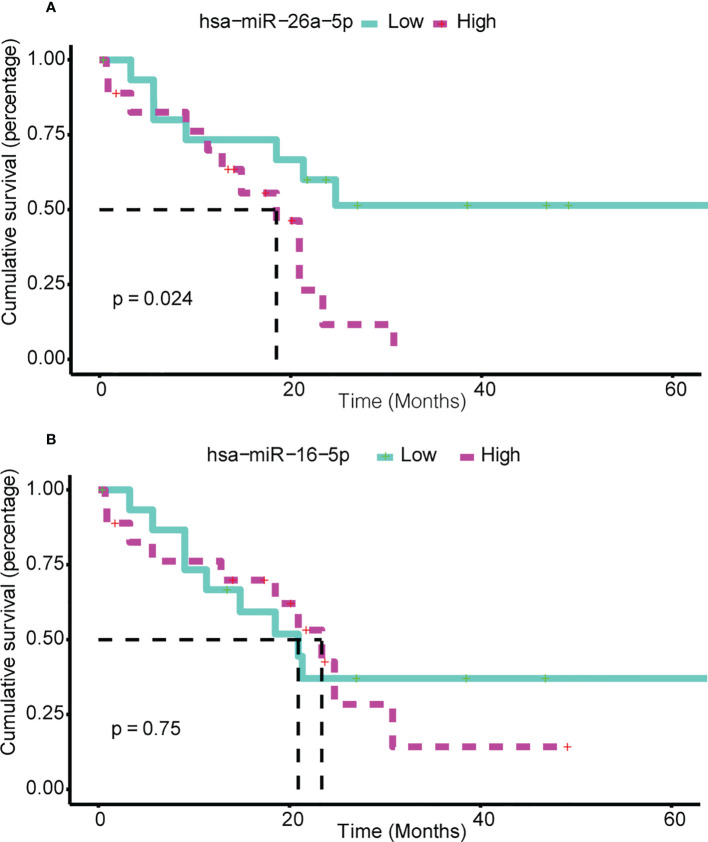
Ser-miRNA was associated with BTC prognosis. **(A)** hsa-miR-26a-5p and **(B)** hsa-miR-16-5p Predict Survival in BTC. The green (red) color bar represents low-express (high-express) patient groups. Kaplan–Meier estimates of 3-year survival of BTC patients in TCGA.

Exhaustive survival analysis was performed on each of the ser-miRNAs in BTC to test whether their expression profiles were associated with cancer prognosis (details in Methods). Numerous studies have evaluated expression profile miRNAs in tissue and serum samples of cancer patients to find appropriate biomarkers for this cancer. We focused on the candidate miRNAs that were common in serum and tissue, and found hsa-miR-16-5p and hsa-miR-26a-5p mapped to the BTC tissues of TCGA. Our data suggested that hsa-miR-26a-5p was a negative prognostic factor in BTC. Using median of hsa-miR-26a-5p expression, patients in the TCGA were divided into high- and low-risk groups; patients with high expression had shorter three-year survival than those with low expression (**Figure 6A**, P = 0.0236). Hsa-miR-16a-5p is not a predictor of three-year mortality (**Figure 6B**, P = 0.75).

### 3.9 Comparison with individual feature selection methods and classifier

The GOADF, SAM, t-test, decision tree fisher and K-Nearest Neighbor (KNN) classifier were compared according to the synergistic AUC for discriminating between BTC patients and healthy controls. GSE85589 was used as the internal dataset, which was randomly divided into an internal training set and an internal test set, and this process was performed 100 times, then we obtained 100 internal training sets and 100 internal test sets. GSE59856 was used as the independent test set.

SAM (P<0.05), t-test (P<0.001), decision tree, fisher and KNN classifier were performed to identify the miRNAs set in each internal training set, and the set contained four miRNAs (**Table S8**). For SAM the best synergistic AUC value was 0.9167 in the internal training set, 0.7619 in the internal test sets, and 0.8790 in the independent test set. For t-test the best synergistic AUC value was 0.9083 in the internal training set, 0.8810 in the internal test set, and 0.9244 in the independent test set. For decision tree the best synergistic AUC was 0.9433 in the internal training set, 0.9167 in the internal test set, and 0.6095 in the independent test set. For fisher classifier the best synergistic AUC was 1 in the internal training set, 0.9750 in the internal test set, and 0.5 in the independent test set. However, the AUC of tree S16L28 was 1 in the internal train set, 0.84 in the internal test set, and 0.9100 in the independent test set. For KNN the best synergistic AUC value was 1 in the internal training set, 0.9762 in the internal test sets, and 0.6627 in the independent test set.

### 3.10 Compared with currently established markers

A search in the National Library of Medicine (https://pubmed.ncbi.nlm.nih.gov) database using the key words ’biliary tract cancer diagnosis serum miRNA’ yielded 19 articles then choose ’early diagonosis’ biomarkers manually, the result displayed serum miR-1281, miR-126, miR-26a, miR-30b and miR-122 were significant differences for between patients with primary sclerosing cholangitis and patients with cholangiocarcinoma (40). Serum miR-122, miR-192, miR-29b and miR-155 were significantly elevated in patients with cholangiocarcinoma compared to healthy controls or patients with primary sclerosing cholangitis without malignant transformation (41), and miR-221 was a biomarker of BTC (42). Mir-744-5p, mir-409-3p, and mir-128-3p were candidate serum miRNAs as potential biomarkers for PC and BTC diagnosis. In addition, the current diagnosis was based mainly on imaging and intraoperative exploration due to having a low sensitivity marker.

There were 5 currently established markers found in the independent test set, and we compared with the biomarkers identified by GOADF in the same data set. The result showed the AUC of currently established mir-744-5p, mir-409-3p, mir-128-3p, miR-1281, miR-30b-3p was 0.6065, 0.5337, 0.5106, 0.5677, 0.7092 respectively for discriminating between BTC patients and healthy controls however, the AUC of hsa-let-7c-5p, hsa-miR-16-5p, hsa-miR-17-5p and hsa-miR-26a-5p were 0.7280, 0.8313, 0.8957, and 0.8303 respectively, and the synergistic AUC was 0.9110. The results showed that the classification performance of the markers we identified is better than the established ones.

## 4 Discussion

BTC is a group of highly aggressive malignant tumors, and histological biopsy is the common diagnostic method for assessing the progression of BTC (43); however, it is invasive and associated with pain. MiRNAs play an important role in the occurrence and development of BTC. It has been reported in the literatures that miRNAs can play a bidirectional role in BTC progression and certain miRNAs can promote cancer progression, while others may have the exact opposite effect, suppressing cancer progression (**Table S9**). Circulating miRNAs have several advantages, for example the detection is minimally invasive, easy to accomplish, and samples can be tested repeatedly (44). Regular monitoring of ser-miRNAs is helpful to detect the progression of malignant tumors and to determine the disease status at an early stage. Therefore, it can provide support for clinical diagnosis and individualized treatment. In addition, circulating miRNAs are widely expressed in cancer and involved in various developmental stages of the tumor (45).

The samples in this study were divided into internal and external datasets, and a resampling technique was used to group samples into pairs of internal training sets and internal test sets (5-fold cross-validation). To explore the classification performance of biomarkers, the training set was divided into a feature selection set and a feature evaluation set (4-fold cross-validation). One sampling produced 20 feature selection sets. Sampling was repeated 100 times at random, and 2000 feature selection sets were produced.

GOADF integrated feature selection methods to optimize key synergistic ser-miRNAs. First, SAM was used to filter ser-miRNAs related to BTC. Then, to identify miRNAs with similar functions, PCC was used to cluster ser-miRNAs into different co-expression sets. To mine key miRNAs that may play an important role in the initiation of BTC and to remove the alternative miRNAs, the t-test was used to select significant representative ser-miRNAs in the co-expression miRNA set. Next, a decision tree was built, and the growth process of the decision tree was the selection process of features. For 2000 feature selection sets, 2000 decision trees were built, which were integrated into a decision forest. Finally, the tree was sorted according to the ST in the forest to obtain the tree with the highest score.

The results showed that the AUC of synergistic ser-miRNAs from a decision tree was higher than that of each single feature in the internal dataset and in the external independent validation set. These synergistic ser-miRNAs are powerful biomarkers for distinguishing PC from BTC. The GOADF was identified as an important tool to detect the synergistic miRNAs essential for the diagnosis of BTC, suggesting that it could be useful for the clinical diagnosis of complex diseases in the future.

We would have liked to use hsa-let-7c-5p, hsa-mir-16-5p, hsa-mir-17-5p, and hsa-mir-26a-5p to classify BTC tissue samples and healthy control samples, but only hsa-mir-16-5p and hsa-mir-26a-5p could be found in tissues datasets. Thus, univariate Cox regression analysis was used to evaluate the association between survival and the expression level of each miRNA, and the result indicated that hsa-miR-16-5p (P=0.0350) was associated with an increased risk of poor survival and hsa-miR-26a-5p (P=0.0980) was not associated with survival.

## Conclusion

In summary, our study developed GOADF to identify four key synergistic ser-miRNAs (hsa-let-7c-5p, hsa-miR-16-5p, hsa-miR-17-5p, and hsa-miR-26a-5p). The synergistic classification performance of the four key synergistic ser-miRNAs was better than the single miRNA’ s for discriminating between BTC patients and healthy controls. This result may provide important insight into the pathogenesis of BTC.

## Data availability statement

Publicly available datasets were analyzed in this study. This data can be found here: https://www.ncbi.nlm.nih.gov/geo/query/acc.cgi?acc=GSE8558; https://www.ncbi.nlm.nih.gov/geo/query/acc.cgi?acc=GSE59856.

## Author contributions

FS developed methodology and directed all the research; ZG: validating the results; YueL: drew figures; GZ and WG analyzed biological significance; CD and YuyL visualization; YiZ, XM and YW analyzed miRNA role; LG analyzed the formal; YaZ wrote the original draft; BL organized the content and structure. All authors contributed to the article and approved the submitted version.

## Funding

This research was funded by the National Nature Science Foundation of China (Young Scientists Fund) (61801151), the China Postdoctoral Science Foundation (2019M651298), the Postdoctoral Foundation of Hei Long Jiang Province (LBH-Z18129, LBH-Z18187), the Natural Science Foundation of Heilongjiang Province (LH2021F053 and H2018014), and the construction project of doctoral program in Traditional Chinese Medicine (Tibetan Medicine) in University of Tibetan Medicine (BSDJS-20-07).

## Acknowledgments

We thank International Science Editing (http://www.internationalscienceediting.com) for editing this manuscript.

## Conflict of interest

The authors declare that the research was conducted in the absence of any commercial or financial relationships that could be construed as a potential conflict of interest.

## Publisher’s note

All claims expressed in this article are solely those of the authors and do not necessarily represent those of their affiliated organizations, or those of the publisher, the editors and the reviewers. Any product that may be evaluated in this article, or claim that may be made by its manufacturer, is not guaranteed or endorsed by the publisher.
